# An Attentional Bias Modification Task, through Virtual Reality and Eye-Tracking Technologies, to Enhance the Treatment of Anorexia Nervosa

**DOI:** 10.3390/jcm12062185

**Published:** 2023-03-11

**Authors:** Franck-Alexandre Meschberger-Annweiler, Mariarca Ascione, Bruno Porras-Garcia, Marta Ferrer-Garcia, Manuel Moreno-Sanchez, Helena Miquel-Nabau, Eduardo Serrano-Troncoso, Marta Carulla-Roig, José Gutiérrez-Maldonado

**Affiliations:** 1 Department of Clinical Psychology and Psychobiology, Institute of Neurosciences, University of Barcelona, Passeig de la Vall d’Hebron 171, 08035 Barcelona, Spain; 2 Department of Population Health Science, University of Utah School of Medicine, 295 Chipeta Way, Salt Lake City, UT 84112, USA; 3 Department of Cognition, Development and Educational Psychology, University of Barcelona, Passeig de la Vall d’Hebron 171, 08035 Barcelona, Spain; 4 Department of Child and Adolescent Psychiatry and Psychology, Hospital Sant Joan de Déu of Barcelona, Passeig de Sant Joan de Déu, 2, Esplugues de Llobregat, 08950 Barcelona, Spain

**Keywords:** body dissatisfaction, body exposure, body-related attentional bias, eye-tracking, virtual reality

## Abstract

Mirror exposure therapies (METs) have been shown to be effective in reducing body image disturbances through the habituation process. Virtual reality (VR) combined with eye-tracking techniques can provide innovative solutions to some of METs’ limitations reported with patients with anorexia nervosa (AN), especially the negative influence of body-related attentional bias (AB). This pilot study aimed to assess the preliminary efficacy of a new VR-based AB modification task (ABMT) among healthy women and the procedure’s user experience. AB levels towards weight- and non-weight-related body parts, using complete fixation time (CFT) and number of fixations (NF), were assessed throughout the ABMT procedure (300 trials). The user experience was evaluated at the end of the procedure. The results showed that VR-based ABMT was effective in reducing AB significantly after 150 trials for both CFT- and NF-based measures, although 225 trials were necessary to get the same result for women with an NF initially more oriented towards weight-related body parts. Overall, the software received a “C-rating” on a scale from “A” (most usable) to “F” (least usable). These results provide evidence of the opportunity to use a VR-based ABMT procedure to reduce AB and improve existing treatments for AN.

## 1. Introduction

Eating disorders (ED) are severe conditions defined by dysfunctional eating behaviors that negatively affect physical and mental health [1]. Anorexia nervosa (AN), characterized by low weight (less than 85% of what is expected considering age and height), alterations in the perception of body image, and an extreme fear of gaining weight, is considered one of the most serious EDs. Indeed, AN has a multitude of medical complications derived from the state of malnutrition and high comorbidity with other disorders, especially anxiety, depressive, and personality disorders [2].

Fear of gaining weight, defined as an extreme fear of the possibility of gaining weight in the entire body or in some specific body parts even at a significantly low weight [3], and body anxiety toward specific body areas (i.e., the body parts that the individuals may relate to weight) are considered one of the strongest risk and maintenance factors of AN symptomatology and have been related to more severe ED symptoms [4,5]. Furthermore, body image disturbances (BIDs) (i.e., the dysfunctional way individuals experience their body weight and shape), both in their perceptual components (body image distortions) and affective components (body image dissatisfaction) (e.g., [6]), cause a series of avoidance behaviors and negative checking strategies towards one’s own body [7,8]. To reduce the effects of these factors, mirror exposure therapies (METs) have been used to enhance AN cognitive-behavioral therapy (CBT) through the habituation process [9,10]. METs, which involve the patients systematically observing their bodies or specific body parts over a certain amount of time and describing them [11], showed promising results in ED patients [12,13] and individuals with high body dissatisfaction [14].

However, the habituation process used in MET protocols might be limited due to cognitive processes leading to selective attention to body information, a phenomenon known as body-related attentional bias (AB). AB is a propensity to pay more attention to certain types of stimuli or information (e.g., disorder-relevant information) than to other sorts of information [8]. Previous studies showed this AB in adult women with high body dissatisfaction (e.g., [15]) and patients with ED (e.g., [16,17]) as a tendency to focus more on self-reported unattractive body parts than other body parts. Indeed, cognitive theories about body dissatisfaction suggest that the processing of information about body image might be influenced by schemas related to appearance, shape, and weight, which lead to increased negative emotions regarding body image (such as fear of gaining weight and body anxiety) and unhealthy behaviors aimed at changing shape and weight for individuals with ED [8].

Furthermore, these theories suggest that body-related AB may be an important risk factor for maintaining BIDs and associated mental health concerns in patients with EDs and healthy individuals (see the full review in [18]). Indeed, many previous studies showed an association between AB and BIDs [18]. For example, when AB was induced toward one’s self-reported unattractive body parts, it led to greater body dissatisfaction in healthy women [19], healthy adolescents, and adolescents with AN and bulimia nervosa (BN) [17]. In contrast, inducing AB toward self-reported attractive body parts of body-dissatisfied women elicits higher levels of body satisfaction (e.g., [14,17]). In addition, an AB predominantly focused on weight-related body parts (such as legs, thighs, buttocks, hips, stomach, or waist) has been shown to be a mediator of the relationship between body mass index and body dissatisfaction [20].

To reduce the negative effects of AB on the mechanisms underlying psychological disorders, AB modification tasks (ABMT) have been proposed to modify early, automatic, and usually unconscious AB [21]. To achieve this, repeated practice of a skill over a period of time was proposed to produce neuroplasticity changes in the brain, in order to strengthen the neural correlates of attention and improve attentional control [22]. Although ABMT procedures have been widely proposed for individuals with anxiety disorders to reduce the attentional avoidance of a threatening stimulus and improve habituation processes (see the overview in [23]), only a few studies have focused on the ABMT procedure in individuals with ED or high body dissatisfaction [22]. Among the studies that proposed ABMT for ED patients or women with high body dissatisfaction, most were focused on food-related AB (e.g., [24,25]). Some of them centered on body-related ABMT but used an attentional probe task to draw attention to negative shape/weight-related words or neutral ones (e.g., [26]). To our knowledge, only two studies have proposed a body-related ABMT procedure using body image stimuli, although with non-clinical participants so far. One study trained attention toward self-reported attractive and unattractive body parts through the eye-tracking (ET) technique [19]. The other was based on the presentation of pictures of the self-defined positive and negative parts of one’s own body using a dot-probe task [27]. Due to a lack of studies so far with ED patients, existing body exposure therapies for the treatment of ED, such as METs, usually aim to extinguish negative cognitive, emotional, and behavioral responses to one’s own body [28], rather than directly modify body-related AB in ED patients [22].

In addition, apart from its limitations due to the influence of body-related AB during the habituation process, METs have other specific limitations with AN patients. First, the risk of eliciting habituation toward extremely low weight and very skinny body shapes may make it difficult to use METs in severe cases of AN [29,30]. METs may increase the probability of rejection and dropout from treatment [9], due to the highly negative reaction of some AN patients while initially observing their bodies [11]. Finally, METs are usually conducted in controlled settings (e.g., therapists’ offices, research laboratories, or ED treatment centers), so it can be difficult to generalize the positive changes learned by applying body exposure protocols [31].

The procedure proposed in the current study, based on new technologies such as virtual reality (VR) and ET, may provide innovative solutions to METs’ limitations mentioned above (the influence of body-related ABs, a high dropout rate, and difficulties in generalizing positive results).

In the last two decades, VR-related hardware and software have made impressive headway, allowing this transformative technology to be used in various fields of psychology, both in research and therapeutics. For example, VR has been extensively and successfully used to investigate and improve exposure-based therapies for the treatment of ED: to propose a food-cue exposure protocol for the treatment of BN and binge-eating disorder (BED) (e.g., [32]), to assess body image distortion and body dissatisfaction [33], to elicit and reduce fear of gaining weight and body anxiety in patients with AN through VR-based body exposure [34], and to modify and improve BIDs in healthy individuals (e.g., [35,36]) and in patients with EDs (e.g., [37,38]). For exhaustive reviews about VR-based therapeutic applications, see [39,40].

VR technology enables researchers and therapists to create highly realistic simulations of real-life settings and situations that individuals have associated with their body and weight concerns (e.g., a dressing room, a bathroom, or a locker room). It also allows the design of three-dimensional (3D) avatars that reproduce the patients’ silhouettes based on their own body size, height, skin tone, and clothes [41]. In addition, VR is capable of moving the same way as individuals due to full-body motion tracking. This encourages participants to perceive and feel their respective virtual bodies as if they were their real bodies by activating the feeling of ownership over a virtual avatar [42,43], a paradigm known as the full-body ownership illusion [44]. As a result, some studies have shown that VR technology could improve treatment adherence and acceptance rates compared to in vivo exposure therapies [45] and have higher ecological validity, allowing generalization of the positive results acquired during VR-based therapies [31,41].

VR technology provides new opportunities for research on AB in patients with ED, due to the integrated ET feature in the Head-Mounted Display (HMD). ET allows a direct, continuous measure of AB that records participants’ saccades toward visual stimuli in real time [46]. By tracking attention over time, ET provides a detailed, direct, and objective picture of attentional patterns, bringing out avoidance and engagement with stimuli over time (e.g., with food cues or the specific body parts of participants). This allows attentional processing to be detected at both automatic and strategic stages. Furthermore, ET-based methods are ecologically valid, as they can be used to study AB on a more naturalistic visual array [47]. Several studies have already used VR technology in combination with ET techniques to investigate AB toward body parts to enhance body exposure techniques for the treatment of ED (full reviews are available in [48,49]). For example, body parts have been classified in weight- and non-weight-related areas based on the delineation established in the physical appearance anxiety scale of the Physical Appearance State and Trait Anxiety Scale (PASTAS) questionnaire [50] to assess AB in both areas of interest and its relationship with ED symptoms (such as fear of gaining weight, body anxiety, or BIDs) (e.g., [51]). The use of these VR and ET techniques revealed differences in the attentional patterns between genders [52,53]. Consequently, the simultaneous use of VR technology and ET techniques offers considerable advantages for developing develop new ABMT procedures, such as greater motivation of the participants to carry out the training, the continuous measurement of attentional patterns during and after the task, or the control of participants’ fixations in real time to ensure that individuals effectively follow the procedure without attentional avoidance.

The current pilot study aimed to evaluate a new body-related ABMT by using VR and ET technologies, among healthy women as a first stage. The proposed procedure was adapted from that of Smeets et al. (2011) [19], in which participants were instructed to detect and identify the nature of geometrical shapes that appeared on their body parts, previously self-reported as “attractive” or “unattractive”. As a result, the attention of the participants was directed to some specific body parts, and AB was temporally modified. The objective of the present study was to assess the preliminary efficacy and usability of this VR-based intervention and figure out the most suitable task duration until a significant reduction in AB was achieved. In addition, the user experience was evaluated at the end of the procedure. The proposed VR-based ABMT intervention was expected to: (i) reduce AB significantly at some point in the procedure compared to the baseline; and (ii) obtain an acceptable user experience rating (i.e., easy-to-use), which would allow a higher level of engagement and a lower dropout rate. Positive results of the present study would enable us, in a second stage, to optimize the procedure and study the efficacy of the ABMT intervention in patients with AN, to reduce AB as a risk and maintenance factor of AN symptomatology, and to enhance possible future therapeutic adaptations to improve currently existing treatments for AN, such as METs or VR-based body exposure.

## 2. Materials and Methods

### 2.1. Participants

Of the seventy women who were initially candidates to participate in the study, six were excluded as they reported that they met at least one exclusion criteria, and four candidates did not complete the full study procedure as they reported dizziness once immersed in the VR environment. Finally, sixty college women (M_age_ = 24.83 years, SD_age_ = 6.64 years; M_BMI_ = 22.23 kg/m^2^, SD_BMI_ = 3.25 kg/m^2^) from the University of Barcelona’s Faculty of Psychology, recruited using social networks and flyers, voluntarily participated in this study and went through the entire procedure. The exclusion criteria were self-reported diagnoses of mental disorders with psychotic or manic symptoms (e.g., psychotic disorders or bipolar disorders), self-reported diagnoses of ED (AN, BN, BED), pregnancy (which could temporarily distort the body image), epilepsy, and visual conditions that could prevent exposure to a VR environment or distort eye-tracking measures.

### 2.2. Instruments

The participants were immersed in a VR-environment using a HMD HTC VIVE Pro Eye™, including dual-OLED displays with a combined resolution of 2880 × 1600 pixels and 615 PPI. The full body tracking feature (i.e., the process of tracing the movements of the participants and applying them to their avatars in real-time within an immersive environment) was ensured using five body trackers: one in the HMD itself, two in the VR controllers the participants hold in their hands, and two feet-trackers (VIVE trackers V3.0), which communicated wirelessly with four SteamVR Base Station 2.0™. This created a sufficient play area (up to 10 m × 10 m) with high stability and pinpoint accuracy. Furthermore, the eye-tracking feature (ET), provided with the HMD HTC VIVE Pro-Eye and powered by Tobii™, was used to detect eye-movement while the participants were looking at their avatars’ bodies in a VR-environment with very high precision (binocular gaze data output frequency: 120 Hz, spatial accuracy between 0.5 and 1.1 degrees, 5-point calibration process).

**Figure 1:**
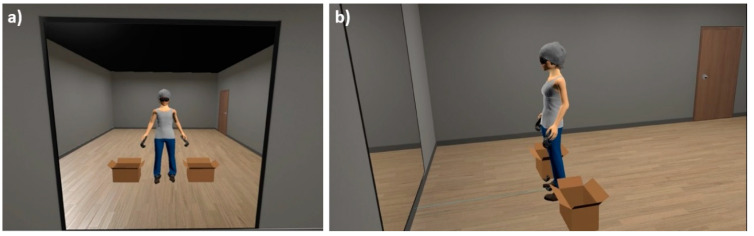
VR immersive environment and female avatar. From left to right: (**a**) participant’s front view; (**b**) profile view.

Each participant was immersed in a VR environment consisting of a room, developed using Unity 3D 5.6.1. software, without any furniture except for a large mirror located 1.54 m in front of the participant and two boxes placed on the floor beside her (see Figure 1). The participant could thus see her whole image in the mirror, even while she was moving, due to full-body tracking. This image consisted of an avatar (see Figure 1), which was initially generated from a generic Caucasian female avatar designed using the software Blender v. 2.78 and then finely adjusted to each participant’s height and silhouette through an initial photography procedure. The avatar wore standard clothes (including a t-shirt, trousers, and shoes, as shown in Figure 1), whose colors could be adapted to fit the actual participant’s clothing. The avatar’s skin color could also be adjusted to fit the participant’s. Finally, to reproduce the actual participant’s condition during the task and to reduce the influence of individual hairstyles, the avatar wore a HMD, and its head was covered by a grey cap.

### 2.3. Measures

#### 2.3.1. Body-Related AB Assessment

**Figure 2:**
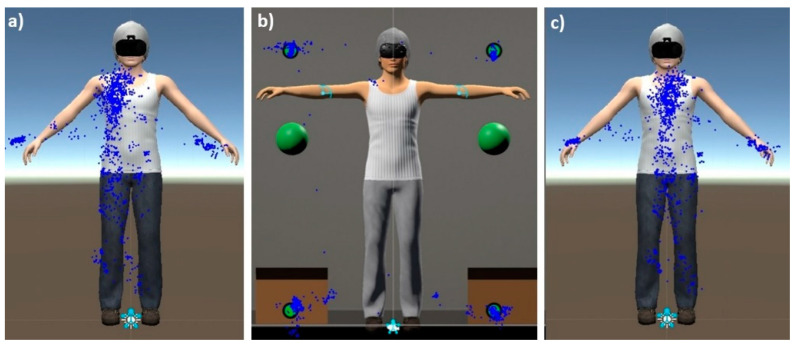
Drift correction procedure. From left to right: (**a**) example of RAW ET data; (**b**) adjustment based on four fixed “drift markers”; (**c**) drift-corrected ET data. Blue dots indicate the fixation pattern of the participant.

To assess the body-related AB, participants were asked to stand with their arms slightly raised and legs separated on fixed reference marks (green spheres for the arms, footprints for the feet), and gaze freely at their avatar in the mirror for 30 s (as done in previous studies, e.g., [51]), while the fixation patterns were recorded by the ET feature of the HMD (RAW ET data). During the process, as a cover story to avoid reactivity, the participant was asked to remain still while the virtual avatar position was recalibrated. In addition, during the process, participants were asked to gaze at four fixed reference marks located around the avatar for 4 s. This information was then used by software developed specifically for this purpose to eventually correct the raw ET fixation data vertically and horizontally (drift-corrected ET data), to validate the ET calibration, and ensure the best precision of the AB assessment (see Figure 2).

The drift-corrected ET data were then imported into the Open Gaze and Mouse Analyzer (OGAMA) software (Freie Universität, Berlin, Germany) to calculate the number of fixations (visual act of maintaining one’s gaze at a single location for a minimum duration, usually 100–200 ms; [54]) and the visual fixation duration in each specific body-related area of interest (AOI). Such specific gaze-behavioral measures have been shown to be a reliable and continuous measure of attention allocation towards specific body areas in previous studies using ET techniques (see full reviews in [47,48]).

**Figure 3:**
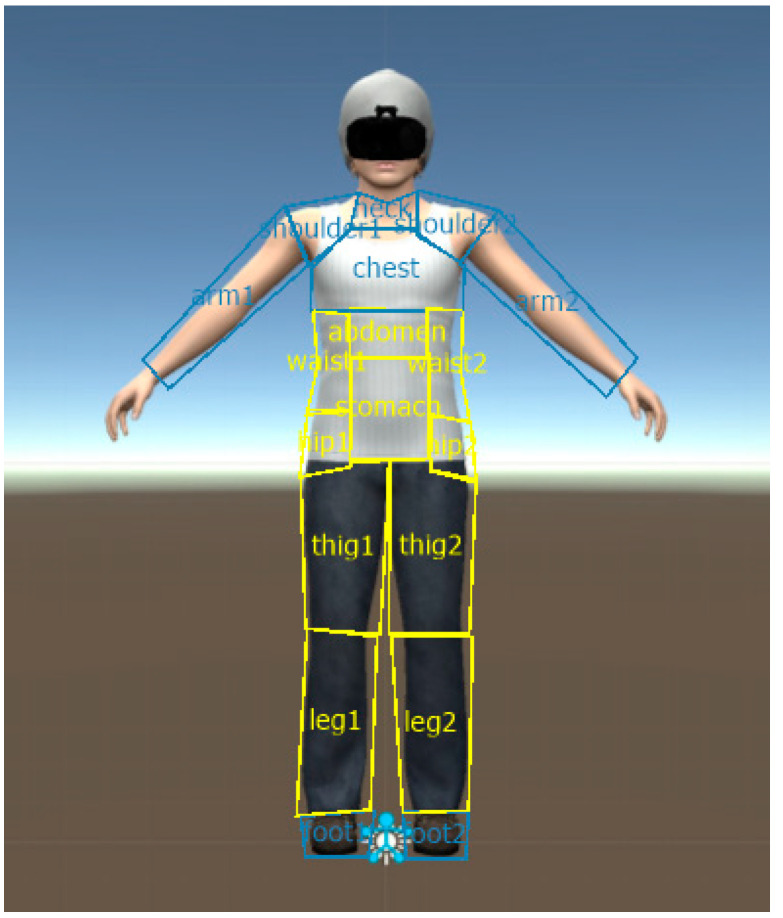
Weight-related and non-weight-related areas of interest (AOIs). Weight-related and non-weight-related areas of interest (AOIs) (yellow and blue areas respectively) in the female avatar, used by the OGAMA software to process body-related attentional bias (both complete fixation time and number of fixations).

AOIs were divided into two groups based on the Weight Scale of body items in the PASTAS questionnaire [50]: the weight-related AOI group, including legs, thighs, buttocks, hips, stomach, and waist; and the non-weight-related AOI group, including the remaining body parts (see Figure 3). The participant’s head was not taken into consideration since the head of the avatar wore an HMD, like the participant, so the fixation of the gaze on this part of the body has more to do with the attention that this device captures than that really dedicated to the head of the participant. The Weight Scale of body items of the PASTAS questionnaire has good internal consistency (with a Cronbach’s alpha of 0.88), a test-retest correlation coefficient (0.89) and convergent validity with other scales of eating disturbances (EDI-DT, EDI-B), body dissatisfaction (EDI-BD), physical appearance evaluation (BSRQ-PAE), and anxiety (STAI) [50].

Finally, like in previous studies (e.g., [55]), AB was assessed through the AB_CFT variable, which is the difference between the complete fixation time (CFT) in the weight-related AOI group and the non-weight-related AOI group, and through the AB_NF variable, which is the difference between the number of fixations (NF) in the weight-related AOI group and the non-weight-related AOI group. Both AB_CFT and AB_NF could thus have positive or negative values, depending on whether the participants’ visual attention was predominantly focused on weight-related AOIs (positive values) or non-weight-related AOIs (negative values).

Only a few studies focused of the reliability and internal consistency of AB measures (such as fixation duration or number of fixations) using the eye-tracking paradigm while participants were freely looking at body parts (mainly emotional faces) (e.g., [56]). Cronbach’s alpha estimates for the total fixation time and number of fixations were high (between 0.94 and 0.96) when participants’ viewing time was greater than 2 s. This indicates the excellent reliability of these measures [56]. However, reliability estimates were very low when the viewing duration was shorter (less than 1 s) but increased afterwards [57]. These results suggest that AB measures using eye-tracking techniques have excellent reliability overall when the measures are averaged over time, but their reliability varies substantially over the presentation time of the display [58]. In the present study, AB was assessed through a 30-s free-viewing task on the avatar in the mirror. In addition, the test-retest reliability of AB measures through ET techniques was estimated to be 0.68 (“good” range) for a one-week test-retest [56] and between 0.39 and 0.65 (“fair” range) for a 6-month test-retest [58]. In the present study, AB assessment’s reliability will be calculated and reported in the “results” paragraph below, using the intraclass correlation coefficient (ICC) that has been shown to be more appropriate than Cronbach’s alpha for ET measurements [59].

#### 2.3.2. User Experience

User experience was assessed through the System Usability Scale (SUS) [60]. The SUS is a 10-item questionnaire with 5 response options. It yields a single number between 0 and 100, which represents a composite measure of the overall usability of the system that is being studied (0 stands for the least usable system and 100 for the most usable one). It is currently the most used questionnaire for measuring perceptions of usability and has been tested on hardware, consumer software, websites, cell phones, interactive voice responses (IVRs), and even the yellow pages. It has become an industry standard, with references in over 600 publications [61]. The scale has good reliability (with a Cronbach’s alpha of 0.91) and concurrent validity (a significant correlation of 0.806 between the SUS and a single 7-point adjective rating question for an overall rating of “user friendliness”) [62].

### 2.4. Procedure

This study was approved by the Bioethics Commission of the University of Barcelona (CBUB). At the beginning of the study, participants freely signed a consent form, which informed them about data confidentiality and the possibility of withdrawing from the study at any point without consequences. Additionally, confidentiality was ensured by assigning a different identification code to each of the participants. Participants were told that the study aimed to study body image disturbances through the virtual reality procedure.

Weight and height were measured to calculate the BMI. A virtual avatar was then created from two photos taken using frontal and lateral perspectives, matching the avatar’s profile to the participant’s real-size body silhouette (e.g., arms, legs, hips, waist, chest, stomach, breast, and shoulders). The participant was then equipped with HTC VIVE Pro Eye™ and body trackers and immersed in the VR room. Once in the room, five-minute visuo-motor and visuo-tactile stimulation procedures adapted from previous studies (e.g., [34]) were applied to elicit a full body ownership illusion (i.e., to perceive and regard a virtual body as one’s own real body). At that point, body-related AB was measured for the first time as the baseline assessment.

Furthermore, the ABMT intervention was applied, following the procedure adapted from Smeets et al. (2011) [19], in which AB was induced toward attractive or unattractive body parts to modify body dissatisfaction among non-clinical female participants. Since the purpose of our study was to reduce AB, the ABMT procedure was aimed at drawing the participant’s attention more equally to all the body parts (i.e., weight- and non-weight-related areas of interest defined based on the Weight Scale of body items of the PASTAS questionnaire [50]).

**Figure 4:**
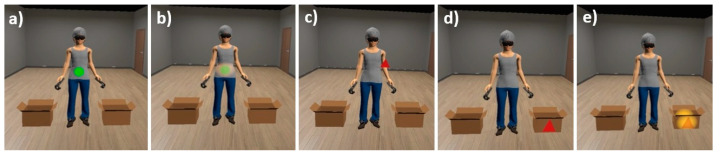
ABMT trials. From left to right: figures appearing on the weight-related body part (stomach): (**a**) initially and (**b**) with illumination on the near-body (while the participant looked at the figure); (**c**) on the non-weight-related body part initially (arm); on the neutral stimulus (box): (**d**) initially and (**e**) with illumination on the area near to the box (while the participant looked at the figure).

Participants were instructed to detect and indicate the shapes (triangles, squares, and circles) or colors (red, green, and yellow) of different geometric figures. The software selected the shape and the color of each figure at random. In each trial, while they were staring at the figures, nearby body areas were lit up progressively. After 4 s holding the gaze, a new figure was projected. If the participant looked away due to a lack of attention before those 4 s, the software waited for them to look at the current projected figure again (the illumination of the nearby body was automatically paused by the software). Real-time fixation data were available due to the HMD’s eye-tracking function. During the intervention, the figures appeared on weight-related body parts in 45% of trials, on non-weight-related body parts in another 45% of trials, and on two neutral stimuli (boxes) located in the ground next to the avatar’s feet for the remaining 10% of the trials (see Figure 4).

The ABMT procedure consisted of projecting a total of 300 figures, divided into 4 series of 75 trials that lasted about 5 to 6 min each. The balanced distribution of figures between weight- and non-weight-related body parts was respected within each series. In addition, figures were equally distributed between right and left sides of the body (in the case of lateralized body parts such as shoulders, arms, legs, and feet). In the first and third series, the participants had to indicate the shape of the projected figure, while in the second and fourth series, they had to indicate the color of the figure. The task for the participants was varied (shapes and colors) to reduce boredom [19]. The required time to complete the task for each series was approximately 6 min.

Immediately after each series, AB was assessed again to provide a total of five AB assessments throughout the intervention (baseline, after series 1, 2, 3 and 4). The participant was allowed a 2-min rest time, sitting in a chair after each AB assessment and before beginning the next ABMT series.

After the last AB assessment, the HMD and body trackers were removed. User experience was assessed using the System Usability Scale Questionnaire. Furthermore, the participant was able to rest for the necessary time while the researcher explained the real objective of the study and answered any possible doubts.

### 2.5. Statistical Analysis

After drift-correction of RAW ET data, the data were imported into the OGAMA software to process both AB variables: AB_CFT (based on the complete fixation time) and AB_NF (based on the number of fixations) for five assessments over time (baseline and after ABMT series 1, 2, 3, and 4). Further details about this procedure can be found in Porras-Garcia et al. (2020) [34]. All the subsequent statistical analyses were performed with SPSS version 27. Two outliers were detected in the AB baseline assessments by inspection of a boxplot. These were excluded from the analysis. Thus, only 58 participants were included (N = 58).

For the purpose of analysis and for each AB variable (AB_CFT and AB_NF), the participants were divided into two AB groups: one group with a positive baseline AB outcome, in which the AB was predominantly oriented towards weight-related body parts, and another group with a negative baseline AB outcome, in which the AB was predominantly oriented towards non-weight-related body parts. The repartition of participants into weight-oriented and non-weight-oriented AB groups was performed independently for each AB variable (AB_CFT and AB_NF), and the statistical analysis was carried out separately.

First, independent-sample *t*-tests were conducted to assess whether there were any significant group differences in age and BMI. Furthermore, mixed between the AB group within AB assessment times, analyses of variance (ANOVA) were conducted for each AB variable (AB_CFT and AB_NF).

Regarding the assumptions of ANOVA analyses, homogeneity of variance and sphericity were met for all AB variables (as demonstrated respectively by Levene’s and Mauchly’s tests with *p* > 0.05). Although some of the AB variables were not normally distributed (as shown by the Shapiro–Wilk tests: *p* < 0.05), it was decided to run the tests anyway since ANOVA is considered a reasonably robust test for deviations from normality [63]. Bonferroni adjustment for multiple comparisons was used in the post-hoc analysis.

As for the user experience, Pearson correlations were run to assess the relationship between the System Usability Scale outcome, BMI, and age in the overall sample.

## 3. Results

First, AB assessment reliability was calculated with SPSS using the intraclass correlation coefficient (ICC) [59]. Both AB_CFT (ICC = 0.619) and AB_NF (ICC = 0.678) showed “good” reliability (between 0.60 and 0.74, following the guidelines for interpretation proposed by Cicchetti, 1994 [64]).

The descriptive results revealed that, overall, participants spent more time looking at non-weight-related body parts than weight-related body parts, as indicated by the negative outcome of AB_CFT (M = −236 ms < 0, SD = 6018 ms). They also showed a higher number of fixations on non-weight-related body parts than weight-related body parts, as indicated by a negative outcome of AB_NF (M = −2.31 < 0, SD = 12.40) (see Table 1).

Independent sample *t*-tests confirmed no significant mean differences (*p* > 0.05) in age and BMI between comparison groups formed from baseline AB assessments. As expected, there were significant mean differences (*p* < 0.001) in baseline AB assessment between comparison groups (i.e., between weight- and non-weight-oriented AB groups), regardless of whether these groups’ formation was done using the complete fixation time or the number of fixations.

**Figure 5:**
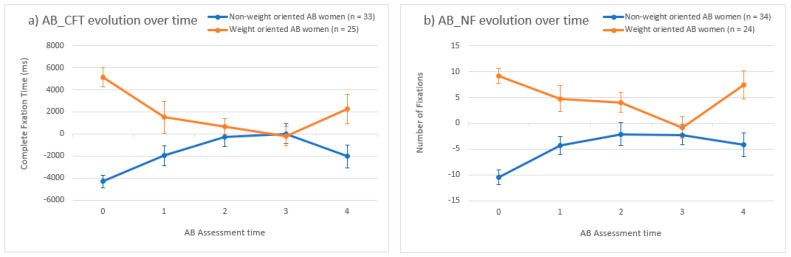
Body-related attentional bias evolution over the intervention. (**a**) Complete fixation time (AB_CFT); (**b**) Number of fixations (AB_NF). Note. Attentional bias (AB) means evolution over time based on (**a**) complete fixation time (AB_CFT); (**b**) number of fixations (AB_NF). ms = milliseconds. AB assessment time: 0 at baseline, 1, 2, 3 and 4 respectively after ABMT series 1, 2, 3 and 4. Error bars represent standard errors of the mean.

Figure 5 shows the evolution of the AB measures (using complete fixation time on Figure 5a and using number of fixations on Figure 5b) over time (during the AB assessment times: at baseline and after each of the 4 ABMT series).

A two-way mixed between AB group and within AB assessment times ANOVA was conducted for both attentional bias measures (i.e., using AB_CFT and using AB_NF). The results indicated a statistically significant interaction between the group and assessment time for the AB_CFT (F (4, 53) = 14.707, *p* < 0.001, partial η^2^ = 0.526) and the AB_NF (F (4, 53) = 7.284, *p* < 0.001, partial η^2^ = 0.355).

Post-hoc analyses assessed the change in AB_CFT values from baseline to each ABMT series separately in both groups. The results revealed that, both in women with a non-weight-oriented AB and in women with a weight-oriented AB, only two ABMT series were necessary to produce a significant (*p* < 0.001) reduction in their AB (see Table 2). Regarding AB_NF, the results revealed that only two ABMT series were necessary to produce a significant (*p* = 0.006) reduction in the AB in women with a non-weight-oriented AB at baseline, while three ABMT series were necessary to produce a significant (*p* = 0.007) reduction in the AB in women with a weight-oriented AB at baseline (see Table 3). Finally, a significant increase in the number of fixations towards weight-related body parts could be noted among weight-oriented AB women after the ABMT fourth series (*p* = 0.030 for AB_NF mean differences between AB assessment after the 3rd ABMT series and after the 4th ABMT series) (see Table 3).

Regarding the user experience, the results with the entire sample (N = 58) of the SUS showed a mean of 67.46 (SD = 10.90) (see Table 1). Pearson correlation analysis showed a significant negative relationship between user experience and age (r(56) = −0.30, *p* = 0.023): the younger the participant, the more usable our software was perceived to be. In contrast, no significant Pearson correlation was found between user experience and BMI (*p* > 0.05).

## 4. Discussion

The results of the pilot study provide initial evidence of the efficacy of the proposed ABMT procedure, through VR and ET technologies, in significantly reducing AB (i.e., obtaining a more balanced attentional pattern between weight- and non-weight-related AOIs), considering the AB_CFT and the AB_NF in healthy women. Furthermore, post-hoc analyses showed that 150 trials (2 series of 75 trials) of the figures’ projection onto the avatar were sufficient to produce a significant reduction in AB_CFT and AB_NF measures, except for the AB processed from the number of fixations among women with initial AB predominantly focused on weight-related body parts. In this last group, 225 trials (3 series of 75 trials) were necessary to obtain the same results. These results are important since body-related AB has been shown to be an important factor in maintaining BIDs in patients with ED [16] and healthy individuals [18] and a mediator of the relationship between BMI and body dissatisfaction [20]. These preliminary results obtained with healthy female participants in this pilot study would have to be confirmed in a future clinical study. They indicate that the proposed ABMT procedure could probably be used as an adjunctive technique to enhance body and mirror exposure therapies for ED patients, whose current objective is to extinguish negative cognitive, emotional, and behavioral responses to their own body [28], considering the modification of the body-related AB underlying the psychological mechanisms involved in these responses [22].

Our study shows the results of AB reduction assessed through two indices (AB_CFT and AB_NF). However, both measures should be interpreted separately, since CFT and NF have been shown to be related to different psychological constructs that reflect and influence cognitive, emotional, and behavioral responses [65]. For example, one study revealed that the nature of the AB can vary over time while emotional pictures are processed. A shorter CFT measured after the first seconds of exposure could indicate avoidance of some detected negative stimuli, even if the NF remains high [66]. Another study with children indicated that increased snack intake was influenced by longer CFT for food cues but not by the NF towards these cues [67]. Possible explanations of these results are that gaze duration activates greater neurological responses (especially in “reward” regions) to food cues than the number of fixations [68]. When fixation time is longer, there is longer activation of brain regions associated with future weight gain and weight maintenance in response to food cues (e.g., middle frontal gyrus, middle temporal gyrus, and insula) [69], or longer activation of brain areas related to food intake regardless of the number of fixations [67]. Thereby, it seems that CFT has a greater influence on emotional, and behavioral responses than NF. However, another study with individuals with depression showed that a greater number of fixations, responsible for repeated turning of attention towards the dysphoric stimuli, rather than longer fixation duration on these stimuli, likely reflects sustained or elaborative processing of the stimuli. Repeated fixations within a stimulus may be necessary to process the image comprehensively [70]. Hence, the choice of 2 series of 75 ABMT trials (required to significantly reduce the AB_CFT for all comparison groups) or 3 series of 75 trials (required to significantly reduce the AB_NF among women with an initial focus predominantly on weight-related body parts) should be carefully considered in future studies, depending on their specific objectives.

This pilot study aimed to obtain preliminary results with non-clinical participants to evaluate the procedure’s efficacy and usability and the ABMT duration that is strictly necessary to ensure this efficacy without producing unnecessary fatigue among participants. Indeed, these results provide researchers with objective data, which allows them to adapt and optimize the procedure in future studies with clinical participants with AN. In fact, standing in front of a mirror and looking at geometric figures for more than 20 min might lead to lower patient engagement and a higher dropout rate. This point is even more important because it is often difficult to keep AN patients committed to treatment, with a high proportion failing to complete the full course of treatment [71]. A significant level of dropout among AN patients, reported in the range of 20–40% for CBT, has serious implications for recovery, research, and the development of new treatments [72]. Several studies have shown that AN patients who leave treatment prematurely are unlikely to recover independently [73] and are more likely to have a poor long-term outcome. In turn, this may lead to greater chronicity of ED symptomatology [74]. For these reasons, it was especially important for us to consider the participants’ user experience throughout the study procedure. Indeed, paying attention to the participants’ own perspective has been highlighted by the increased focus on assessing client satisfaction in ED health services [75,76], since dissatisfaction with treatment can lead to treatment delay, failure to engage, and, ultimately, treatment withdrawal [77,78].

Regarding the procedure’s usability, our results indicated that the user experience was very close to the average SUS score of 68 calculated from 500 studies, granting our software a “C-grade” on a scale ranging from “A” (most usable) to “F” (least usable) [61], with a more satisfying user experience reported by younger participants. These results are acceptable as a starting point, but special attention will have to be paid to improving the user experience, especially considering the age and BMI of the participants in future clinical studies with AN patients. Indeed, our sample had a mean age of 24.93 years (with SD = 6.73 years) and a mean BMI of 22.28 kg/m^2^ (with SD = 3.27 kg/m^2^). In contrast, 40% of individuals with AN are diagnosed during early adolescence or adolescence [79,80], and their BMI should be less than 85% of what would be expected considering age and height [1]. A previous study showed that age influences body representations and thus the user experience, as older participants (more than 26 years old) were more resistant to changes induced by the bodily illusion than younger participants [81]. A better user experience reported by younger individuals in our study may thus positively influence the efficacy of the proposed VR-based ABMT procedure among younger participants (which is often the case for AN patients). For these reasons, it might be recommended that future research assess the efficacy of the proposed intervention separately for adults and adolescents with AN.

Regarding the ABMT’s duration, the results of the AB assessment after the fourth ABMT series revealed that extending the task to more than 225 trials might be counterproductive. Indeed, the results showed that the mean differences in AB measures increased between the evaluation times after the third and fourth series in both comparison groups and in both AB indices (AB_CFT and AB_NF). However, this increase was only statistically significant for the group of women with an initial AB_NF orientation toward weight-related body parts. It is difficult to interpret this outcome with the available data, but this may be due to a certain relaxation in the participants’ attention during the last AB assessment task since they knew at that moment that the procedure was about to end. Another possible explanation of these results would be that, after 3 ABMT series (225 trials lasting around 18 min), the participants felt a greater level of stress and/or fatigue, which negatively affected their attention during the fourth series and the last AB assessment task. Indeed, the proposed VR-based ABMT procedure required both selective attention, which involves processing parts of the sensory input to the exclusion of others (i.e., in this study, locating and indicating each of the 300 geometric figures projected on the avatar), and sustained attention, which involves maintaining sensitivity to incoming stimuli over a long period of time (i.e., in this study, maintaining attention during a fairly long exposure procedure, as it took approximately 24 min to complete all four ABMT series). A previous study revealed that attentional performance (especially in its visual modality, not so much in its auditory one) could be negatively affected due to the increase in the level of stress over time [82]. The qualitative verbal feedback given by the participants during the debriefing at the end of the procedure may confirm such a hypothesis, since they often mentioned that the task was “long”, “repetitive” and “boring”. Hence, taking into account the above considerations and in order to ensure a proper balance between efficacy (i.e., a significant reduction in AB) and usability (getting participants committed to a “user-friendly” and “easy-to-use” procedure), we would recommend not extending the ABMT procedure to more than 225 trials: ideally 150 trials (considering AB_CFT assessment) or 225 trials (considering AB_NF assessment). Such a reduction in body-related ABMT, if confirmed in a further clinical study, could then be used in clinical practice to improve the efficacy of the usual AN treatment (e.g., mirror exposure therapy) carried out immediately after this AB modification.

In the current study, the body-related AOIs used during AB assessment and ABMT intervention were defined based on the weight-related scale of the PASTAS questionnaire [50] and were the same for all participants without considering individual differences. Such a methodology differs from other studies, using a similar free-viewing and single-body paradigm but measuring the AB toward self-reported attractive vs. unattractive body areas (e.g., [14,17]). Since there may be weight- or non-weight-related body areas in both self-reported attractive and unattractive body parts for a specific participant, such differences may influence the results of the present study compared to other studies using other body-related AOI classification methodologies. Even so, the use of the weight-related scale of the PASTAS questionnaire, statistically representing higher body dissatisfaction (significant positive correlation between the weight-related scale of the PASTAS and body dissatisfaction assessed through the EDI-BD questionnaire [50]), has the advantage of simplifying the process in comparison with self-reported attractive vs. unattractive body areas. In addition, it is important to take into account that this study only included healthy participants, whose attentional patterns may be different than those of clinical patients with AN. Indeed, a previous study showed that AN patients have a longer fixation time and more fixations on weight-related body parts than non-clinical individuals [55]. Hence, the efficacy of the proposed ABMT procedure cannot be generalized and will have to be investigated in further clinical studies.

However, some limitations in VR software should be considered in order to be addressed in future studies. First, the VR software allowed adaptation of the color of the avatar’s clothing and skin tone to match the actual appearance of each participant as closely as possible. However, although the general silhouette of the virtual body respected that of the participant (thanks to the initial photographic procedure), some specific parts of the avatar were not exactly that of each individual, such as the hairstyle (covered by a grey cap in the VR environment), the facial lines (most of the face was hidden by black glasses that simulated the HMD), or the specific outfit of each participant at the time of the intervention (the type of the avatars’ t-shirt, trousers, and shoes were the same for all participants). Hence, the full body ownership illusion (i.e., the identification with the virtual body) could be limited [83], which in turn could decrease the efficacy of the intervention. The use of 3D body scanning, allowing the simulation of an exact 3D biometric avatar with all the individual’s features, would notably enhance the realism of VR embodiment-based techniques and, consequently, improve VR studies on body-related issues for ED treatments. Some recent studies have already benefited from this 3D scanning technology to create more realistic avatars (e.g., see [84]). However, such technology is not usually available in eating disorder treatment centers (mainly due to its high cost), so our protocol of creating avatars of participants based on a photography procedure may be more adapted to clinical practice. In addition, the VR immersive environment generated by our software was quite neutral (only furnished with a mirror, a door, and two boxes; see Figure 1) and did not correspond to other settings that are more relevant in everyday life situations (e.g., a dressing room or bathroom). Consequently, the improvement in the VR environment in future research would be an opportunity to enhance the ecological validity of the studies, taking full advantage of all the possibilities offered by VR technology.

In summary, despite its limitations, this study shows promising results for the reduction in body-related AB using VR technology and ET techniques. Future research should test the implications of the current findings in clinical cases of AN using procedures that include multiple sessions of ABMT as an adjunctive treatment to METs and/or CBT and a follow-up evaluation of the efficacy of the intervention in the longer term.

## Figures and Tables

**Table 1:** Descriptive results: mean (M) and standard deviation (SD).

	Overall (N = 58)	Grouping According to Complete Fixation Time		Grouping According to Number of Fixations	
		Weight-Oriented AB_CFT Women (n = 25)	Non-Weight-Oriented AB_CFT Women (n = 33)	Weight-Oriented AB_NF Women (n = 24)	Non-Weight-Oriented AB_NF Women (n = 34)
	M (SD)	M (SD)	M (SD)	M (SD)	M (SD)
Age	24.93 (6.73)	25.48 (7.62)	24.52 (6.06)	24.04 (2.46)	25.56 (8.54)
BMI	22.28 (3.27)	22.67 (3.80)	21.99 (2.82)	22.63 (3.71)	22.03 (2.95)
Baseline AB_CFT ***	−236 (6018)	5135 (4404)	−4305 (3219)	4606 (5063)	−3654 (3952)
Baseline AB_NF ***	−2.31 (12.40)	7.72 (8.22)	−9.91 (9.23)	9.17 (7.00)	−10.41 (8.26)
SUS	67.46 (10.90)	65.70 (10.47)	68.79 (11.18)	65.62 (10.51)	68.75 (11.13)

Note: body mass index (BMI); attentional bias computed from complete fixation time in milliseconds (AB_CFT); attentional bias computed from number of fixations (AB_NF), user experience through the system usability scale (SUS). Independent sample *t*-tests between comparison groups are significant if *** (*p* ≤ 0.001).

**Table 2:** Post-hoc analyses: pairwise comparisons-AB_CFT Mean differences over time for each group.

AB_CFT Assessment Times		Weight-Oriented AB_CFT Women (n = 25)		Non-Weight-Oriented AB_CFT Women (n = 33)	
(I) Time	(J) Time	MD (I-J) (ms)	95% CI	MD (I-J) (ms)	95% CI
0	1	3616.04	(−347.96, 7580.04)	−2334.70	(−5784.92, 1115.52)
	2	4453.32 ***	(1434.96, 7471.68)	−4045.33 ***	(−6672.48, −1418.19)
	3	5330.64 ***	(2178.41, 8482.87)	−4326.00 ***	(−7069.67, −1582.33)
	4	2881.80	(−892.14, 6655.74)	−2291.06	(−5575.86, 993.74)
1	2	837.28	(−2964.86, 4639.42)	−1710.64	(−5019.98, 1598.70)
	3	1714.60	(−2769.44, 6198.64)	−1991.30	(−5894.16, 1911.55)
	4	−734.24	(−5283.27, 3814.79)	43.64	(−3915.78, 4003.06)
2	3	877.32	(−2555.24, 4309.88)	−280.67	(−3268.33, 2706.99)
	4	−1571.52	(−5326.34, 2183.30)	1754.27	(−1513.88, 5022.43)
3	4	−2448.84	(−6243.37, 1345.69)	2034.94	(−1267.77, 5337.65)

Note. AB_CFT mean differences (MD) and 95% confidence intervals (CI) stated for each group comparison. ms = milliseconds. AB_CFT assessment times: 0 at baseline, 1, 2, 3 and 4 respectively after ABMT series 1, 2, 3 and 4. Adjustment for multiple comparisons: Bonferroni. The mean difference is significant if *** *p* ≤ 0.001.

**Table 3:** Post-hoc analyses: pairwise comparisons-AB_NF Mean differences over time for each group.

AB_NF Assessment Times		Weight-Oriented AB_NF Women (n = 24)		Non-Weight-Oriented AB_NF Women (n = 34)	
(I) Time	(J) Time	MD (I-J) (ms)	95% CI	MD (I-J) (ms)	95% CI
0	1	4.42	(−3.45, 12.28)	−6.09	(−12.70, 0.52)
	2	5.12	(−2.82, 13.07)	−8.32 **	(−15.00, −1.65)
	3	10.00 **	(1.84, 18.16)	−8.18 **	(−15.03, −1.32)
	4	1.75	(−7.28, 10.78)	−6.24	(−13.82, 1.35)
1	2	0.71	(−8.14, 9.56)	−2.24	(−9.67, 5.20)
	3	5.58	(−2.78, 13.94)	−2.09	(−9.11, 4.94)
	4	−2.67	(−10.90, 5.57)	−0.15	(−7.07, 6.77)
2	3	4.87	(−3.06, 12.81)	0.15	(−6.52, 6.81)
	4	−3.37	(−12.19, 5.44)	2.09	(−5.32, 9.49)
3	4	−8.25 *	(−16.03, −0.47)	1.94	(−4.59, 8.48)

Note. AB_NF mean differences (MD) and 95% confidence intervals (CI) stated for each group comparison. ms = milliseconds. AB_NF assessment times: 0 at baseline, 1, 2, 3 and 4 respectively after ABMT series 1, 2, 3 and 4. Adjustment for multiple comparisons: Bonferroni. The mean difference is significant if * *p* ≤ 0.05, ** *p* ≤ 0.01.

## Data Availability

The data presented in this study are available on request from the corresponding author.
